# Timing matters? The effects of two different timing of high protein diets on body composition, muscular performance, and biochemical markers in resistance-trained males

**DOI:** 10.3389/fnut.2024.1397090

**Published:** 2024-05-23

**Authors:** Mohammadyasin Lak, Reza Bagheri, Hamid Ghobadi, Bill Campbell, Alexei Wong, Amin Shahrbaf, Mohammad Shariatzadeh, Fred Dutheil

**Affiliations:** ^1^Sport Sciences Research Institute of Iran, Tehran, Iran; ^2^Department of Exercise Physiology, University of Isfahan, Isfahan, Iran; ^3^Department of Exercise Physiology, Ferdowsi University of Mashhad, Mashhad, Iran; ^4^Performance and Physique Enhancement Laboratory, University of South Florida, Tampa, FL, United States; ^5^Department of Health and Human Performance, Marymount University, Arlington, TX, United States; ^6^Faculty of Medicine, Shahid Beheshti University of Medical Sciences, Tehran, Iran; ^7^Université Clermont Auvergne CNRS, LaPSCo, Physiological and Psychosocial Stress, CHU Clermont-Ferrand, University Hospital of Clermont-Ferrand, Preventive and Occupational Medicine, Clermont-Ferrand, France

**Keywords:** exercise, dietary protein, nutrition, muscle hypertrophy, strength

## Abstract

**Background:**

It is unclear whether resistance training in combination with different timing of protein intake might have differential effects on muscle hypertrophy, strength, and performance. Therefore, we compared the effects of 8 weeks of resistance training combined with two different high-protein diet strategies (immediately pre-and after, or 3 h pre and after exercise) in resistance-trained males.

**Methods:**

Forty resistance-trained males (24 ± 4 years) performed 8 weeks of resistance training combined with 2 g kg^−1^ d^−1^ protein. Body composition, muscular performance, and biochemical markers were assessed pre and post-intervention.

**Results:**

Nine participants (four from 3 h group and five from the immediate group) withdrew from the study. Therefore, 31 participants completed the study. All measures of skeletal muscle mass, Australian pull-up, and muscle strength, significantly increased post-intervention in both groups (*p* < 0.05). The biochemical marker urea also significantly increased from pre to post in both groups (*p* < 0.05). There were no significant between-group differences (*p* > 0.05).

**Conclusion:**

High-protein diet enhances muscular performance and skeletal muscle mass in resistance-trained males, irrespective of intake time. Consequently, the total daily protein intake appears to be the primary factor in facilitating muscle growth induced by exercise.

## Introduction

For individuals looking to optimize improvements in muscle hypertrophy and strength, participation in a resistance training program in conjunction with adequate dietary protein intake is necessary (1, 2). Key variables in resistance training that are often manipulated to maximize muscle hypertrophy include training volume, intensity, and frequency (3). Similarly, there also exists dietary protein intake variables to maximize training adaptations, which include total daily protein intakes, within-day protein distribution (relationship between the quantities of dietary protein consumed meal to meal to the overall intake), and the timing of protein intake relative to the resistance exercise stimulus (i.e., protein timing) (4, 5).

Relative to total daily protein intakes, higher protein intakes are superior to lower protein intakes (1, 6–8). A threshold for total daily protein intake has been reported to be approximately 1.6 g kg^−1^ d^−1^ for individuals looking to improve hypertrophy outcomes (1, 6, 9). Protein intakes surpassing this threshold are not likely to provide further benefit relative to gains in skeletal muscle mass (SMM) or strength (10, 11). The distribution of dietary protein intake throughout the day also represents an important consideration for optimizing protein intake in resistance-trained athletes. Prior work in this area has reported consuming a balanced distribution of protein throughout the day, consisting of 4–5 evenly spaced feedings to be optimal for maximizing post-prandial muscle protein synthesis (MPS) rates and muscle hypertrophy outcomes (12, 13).

While there appears to be a consensus with respect to total daily protein intakes and the distribution of that protein to optimize skeletal muscle hypertrophy adaptations to resistance training, the effect of the timing of protein intake around exercise on hypertrophy outcomes remains unclear, with some research reporting improvements in muscle hypertrophy with protein timing and others concluding no hypertrophic benefits associated with protein timing. In particular, previous work has found notable improvements in muscle size, lean mass, and strength when protein is consumed within a specific window of time before and/or after a bout of resistance training in younger, trained individuals (14). However, other studies conducted in similar populations have found little to no effect of protein timing (15). There have also been two systematic reviews and meta-analyses on protein timing, with both reporting no beneficial effect of a specific timing of protein intake on muscle hypertrophy (16, 17). However, the majority of studies included in previous systematic reviews and meta-analyses assessing the impact of protein timing on changes in muscle size and strength had an inactive comparator design (i.e., while the intervention group was given a protein supplement before and/or after exercise, the control group was not provided a comparable dose of protein at any point during the intervention). Stated differently, most studies included in systematic reviews and meta-analyses of protein timing lacked a control group with matched protein type, dose, and frequency, thereby limiting the conclusions drawn about protein timing. This is a major weakness in this body of research that needs to be addressed. Given these methodological limitations, more research into this protein timing is warranted.

This present study aims to unravel the intricate relationship between protein timing and its effects on muscle performance and body composition among resistance-trained males. Through a randomized clinical trial, we seek to investigate the contrasting impacts of protein intake 3 h before and three hours after exercise, compared to the immediate intake of protein before and after a resistance training session. By examining the differences between these timing strategies, we hope to provide empirical insights into the most effective approach for optimizing muscle growth, recovery, and body composition in this population. Such insights are crucial for refining dietary protein intake in conjunction with resistance training protocols, thereby optimizing the training adaptations of dedicated male athletes striving to achieve their peak potential.

## Methods

### Participants

Forty resistance-trained males (24 ± 4 years) were recruited for this study from November 2022 to March 2023, following their presentation at our physical and sports medicine office. Before the initial assessment, nine individuals dropped out for personal reasons. The inclusion criteria comprised performing resistance training at least three times a week for 1 year prior to the start of the study, not taking any steroids or supplements for at least 1 year prior to the start of the study, having no proven medical issues or musculoskeletal disorders, abstaining from alcohol and tobacco, sleeping for at least seven to 8 h during the 24 h day, and having a protein intake lower than ~2 g kg^−1^ d^−1^. Possible participants were excluded from participating through failure to meet any of the previously stated criteria. Additional exclusions were non-willingness to continue protein intake or performing exercise protocols, participation in other additional exercises than the prescribed resistance training program, consumption of dietary supplements (other than the protein intervention) during the study period, and missing more than one training session or protein intake throughout the study. A physician using the Physical Activity Readiness Questionnaire (PAR-Q) and medical health/history questionnaire conducted the eligibility assessment. Participants deemed eligible provided written and verbal consent. All protocols were approved by the Research Ethics Committees of the Sport Sciences Research Institute, Tehran, Iran, carried out in accordance with the Declaration of Helsinki and registered at clinicaltrials.gov (NCT05544955).

### Study design

This investigation constituted a randomized clinical trial employing convenience sampling methodology. The randomization was conducted through the block randomization method, based on a 1:1 equation. A matched-pair design was used to facilitate the allocation of participants into experimental groups. Initially, participants were ranked according to their SMM, arranged from highest to lowest. Subsequently, the ABBA assignment procedure was applied for group allocation. This approach involves assigning the participant with the highest SMM score to group A, followed by the placement of the next two highest-scoring participants in group B. This pattern is repeated (fourth and fifth highest in group A, sixth and seventh in group B, and so on) until all participants are allocated. This alternating assignment pattern ensures a balanced distribution, where for each sequential pair of participants (1 and 2, 3 and 4, 5 and 6, etc.), both groups receive alternately either the higher or lower SMM score within each pair, thus maintaining an equitable group composition (18).

The trial began with 40 participants, evenly randomized into two intervention groups, either resistance training +2 g kg^−1^ d^−1^ of protein 3 h prior to and 3 h after (Please see “diet” section for more details) resistance training (3 h; *n* = 20) or resistance training +2 g kg^−1^ d^−1^ of protein immediately prior to and after resistance training (immediate; *n* = 20). Both groups engaged in an 8 weeks resistance training regimen, consuming 2 g kg^−1^ d^−1^ of protein, of which 50 g of protein was concentrated and isolated whey protein on training days. On non-training days, the total protein intake was consumed through the daily diet. Prior to baseline measurements, participants underwent familiarization with all testing and experimental procedures. Data collection (body composition, muscular performance, and blood sampling) occurred at two time points—at baseline and 8 weeks post-resistance training. Final assessments were conducted approximately 72 h after the last exercise session to mitigate any acute effects of resistance training on outcome variables. Consistency in measurement conditions was maintained, with all recordings taken at the same time of day (within ~1 h) and under the same environmental conditions (approximately 20°C and 55% humidity). All outcome assessors were blinded to group assignments. Participants maintained food records throughout the study to facilitate the quantitative analysis of total energy (kcal) and macronutrient intake over time. Throughout the study duration, participants were advised to maintain their regular lifestyle (other than their assigned intervention).

### Anthropometry and body composition

The participants were provided with instructions to ensure they arrived at the laboratory in a state of hydration and after having fasted overnight (it was consistent on pre-and post-testing days). Moreover, participants followed a uniform diet regimen preceding testing sessions, and a 24 h dietary recall was collected from each participant. In order to prevent errors in assessing hydration status, participants were instructed to completely empty their bladders upon entering the laboratory. Additionally, they were advised to refrain from consuming beverages containing caffeine, alcohol, and other substances having diuretic properties for a period of 12 h preceding the measurements. The participants’ body mass was assessed using a digital scale manufactured by Lumbar in China, with measurements recorded to the nearest 0.1 kg. Height measurements were obtained using a stadiometer produced by Race Industrialization in China. The study used a multi-frequency bioelectrical impedance equipment, namely the Inbody 770 from South Korea, to assess several body composition parameters including SMM, fat mass (FM), and body mass index (BMI). Prior to the measurement, the palms and soles of the participants were cleansed using an electrolyte tissue. The participants then positioned themselves on the InBody 770 device, ensuring that the soles of their feet were in contact with the electrodes. The instrument obtained the body mass of the participants, while the researcher manually entered their age and sex into the display. The participants proceeded to firmly hold the handles of the device, ensuring that the palm and fingers of each hand established direct contact with the electrodes. They maintained their arms in a fully extended position, with an abduction angle of about 20°. Analysis of body composition was determined by the unit with participants remaining as motionless as possible (19). The bioelectrical impedance approach has good test-retest reliability (*R* = 0.96 to 0.98).

### Maximal strength

The assessment of maximal strength was conducted by using the one-repetition maximum (1-RM) test for both the leg press and chest press exercises following guidelines by the National Strength and Conditioning Association (20). These measurements were then used to predict the appropriate training intensity levels for resistance training regimens. Prior to beginning the examination, the researchers provided a comprehensive overview of the objectives, potential hazards, discomforts, responsibilities of the participants, advantages, inquiries, and consent. Prior to the commencement of the testing session, the participants were instructed to refrain from alcohol consumption for a duration of 48 h, avoid the intake of caffeinated drinks for a period of 12 h, and abstain from eating meals for a span of 2 h. The drinking of water was authorized. The participants thereafter conducted two trials, recording their maximum weight lifted and the total number of repetitions performed. The total number of repetitions necessary to achieve a state of exhaustion did not exceed 10. The participants were provided with a designated period of three to five minutes of rest between each trial, during which no external stimuli were presented. Using the formula 1-RM = weight/(1.0278–0.0278 repetitions) (21), the maximum strength of participants was predicted after the testing session.

### Muscular endurance

Following the completion of the 1-RM in the morning, participants were provided with instructions to engage in leg-and chest press exercises at 75% of their 1-RM. (1) The purpose of this exercise was to assess muscular endurance, which was measured by the number of successful repetitions performed prior to reaching technical failure. Technical failure was defined as the point at which participants were unable to execute another repetition with proper form. This assessment took place in the evening (22).

### Performance testing

The assessment included measuring the maximum height achieved during the vertical jump and the total number of Australian pull-ups completed in a single set. Typically, every participant engaged in a warm-up routine that included a 5 min run or bike session on a treadmill or cycle ergometer, performed at a self-determined leisurely intensity. This was followed by a dynamic warm-up comprising 10-yard repetitions of high knees, butt kicks, side shuffles, and karaoke running drills. Finally, the warm-up concluded with 10 repetitions of pushups and 10 repetitions of bodyweight squats. The participants thereafter engaged in a period of rest lasting 3–5 min before starting the performance tests. Consequently, the following tests were conducted in the prescribed sequence: the vertical jump test, whereby the highest value was recorded based on a maximum of three tries; and the Australian pull-up test, wherein the greatest number of repetitions was recorded based on a maximum of three attempts (1). In all experiments, a rest period of roughly 5 min was implemented.

### Blood tests

Blood samples were collected from the cubital vein using conventional protocols after an overnight fast of 8 h. The samples, measuring 5 mL, were obtained at the same time of day for both pre-and post-testing. Liver enzymes [alanine transaminase (ALT; intra-assay CV: 1.81%; inter-assay CV: 2%)], aspartate aminotransferase (AST; intra-assay CV: 2.01%; inter-assay CV: 2.54%), and gamma-glutamyl transferase (GGT; intra-assay CV: 1.56%; inter-assay CV: 0.92%), creatinine (intra-assay CV: 1.60%; inter-assay CV: 2.24%), and Urea Nitrogen (BUN) (intra-assay CV: 2.20%; inter-assay CV: 3.36%), were measured in serum. Liver and kidney function markers and lipid profiles low-density lipoprotein (LDL; intra-assay CV: 0.64%; inter-assay CV: 1.37%), high-density lipoprotein (HDL; intra-assay CV: 0.77%; inter-assay CV: 1.80%), cholesterol (intra-assay CV: 1.11%; inter-assay CV: 1.18%) were measured in duplicate using Pars Azmoon kits and the spectrophotometric method (DiaSys Diagnostic Systems GmbH, Germany) after 48 h following the last training session.

### Resistance training and protein intake procedures

The resistance training protocol in this study was subject-specific. Based on each participant’s self-reported training volume (reported at the baseline), participants conducted a four-days of resistance training sessions per week (for those who had a volume of <20 sets per week) or a five-days of resistance training sessions per week (for those who had a volume of >20 sets per week) (23). The four-day regimen encompassed sessions targeting the upper and lower body (two sessions each), while the five-day regimen incorporated an additional upper body session (3 days of upper body and 2 days of lower body). The training protocol adhered to a non-linear periodization model, with repetition ranges predominantly spanning 8–15 per exercise, maintaining reps in reserve (RIR) of 1–2. The periodized resistance training program was adapted from previous literature (23). All training sessions were performed under the supervision of a Certified Strength and Conditioning Specialist. If subjects missed a scheduled training session, a makeup session was performed within a week (24). Also, resistance training volume was calculated using the following formula in each session and was reported weekly (25): Resistance training volume = [repetitions (*n*) × sets (*n*) × load or selected weight (kg)].

### Diet

Participants completed six 24 h dietary logs (4 non-consecutive weekdays and 2 non-consecutive weekend days) to determine habitual protein intakes. To assist in achieving their targeted protein intake (i.e., 2 g kg^−1^ d^−1^) (26), participants consumed 50 g of concentrate and isolated whey protein (ISS nutrition, Iran) beverage prior to (25 g of protein) and upon cessation (additional 25 g of protein) of every training session that comprised the following nutrition profile per scoop (33 g): calories, 126; total fat, 1.8 g; saturated and trans-fat, sugars and dietary fiber, 0 g; sodium, 50 mg; potassium, 112 mg; total carbohydrate, 2.8 g; protein, 25 g. Other remaining protein quantities were consumed via foods, and habitual dietary protein intake remained stable throughout the intervention for both groups. Participants attended consultations with an accredited practicing dietitian every week, where they were provided guidelines to reach protein and energy needs, including the distribution of protein intake throughout the day across 4–7 meals with 20–40 g of protein per meal to maximize MPS (27, 28). Macronutrient composition was supervised during the study, with total energy intake (TEI) and protein intake a focus. Carbohydrate and fat intake were suggested to be within the Acceptable Macronutrient Distribution Range for these macronutrients (45–65% and 20–35% TEI for carbohydrate and fat, respectively). Food records were kept daily by participants throughout the study using mobile phone applications MyFitnessPal^®^ or Karafs^®^ applications. All dietary intake data were analyzed using (Diet Analysis Plus, version 10; Cengage) to ensure the same food database was used for all analyses.

### Statistical analysis

The sample size calculation was carried out using the G-power 3.1.9.2 software, based on *a priori* calculations. The justification for determining the sample size was predicated on our prior research, which substantiated notable enhancements in lean mass subsequent to a high-protein diet combined with resistance training in trained males (2). By utilizing the equation for effect size (ES) [(mean before-mean after the high protein diet)/the pooled standard deviation], this study revealed an ES of 0.28 [(53.1–51.7)/4.88]. In the present study, based on *α* = 0.05, a power (1 − β) of 0.80, and an ES = 0.28 (highest approximate effect size), a total sample size of at least 28 participants (*n* = 14 per group) was needed for sufficient power to detect significant changes in the primary outcome of SMM. The normality of the distribution of all variables was evaluated before performing statistical analyses using the Shapiro–Wilk test; there were no missing values at any time point. Baseline characteristics (at PRE) between groups were reported using mean ± SD and examined using independent *t*-test. Effects of training and nutritional interventions on dependent variables were analyzed using a two × two analysis of variance (ANOVA) with repeated measures [time (pre-test vs. post-test) × group (3 h vs. immediate)] to determine the differences between the treatments over time. When the group-by-time interaction was significant, the Sidak multiple comparison test was used to determine between-group differences. Pearson’s simple linear regressions and correlations were performed with a 95% confidence interval (CI). Values between 0 and 0.3 (0 and −0.3) indicate a weak positive (negative) linear relationship through a shaky linear rule. Values between 0.3 and 0.7 (−0.3 and −0.7) indicate a moderate positive (negative). Values between 0.7 and 1.0 (−0.7 and −1.0) indicate a strong positive (negative) (29). All analyses and figure production were performed using GraphPad Prism (version 8.4.3).

## Results

### Participant characteristics

Initially, 40 healthy resistance-trained males were allocated to our study groups. However, nine participants (four from 3 h group and five from the immediate group) withdrew from the study due to personal reasons and musculoskeletal injuries. There were no significant between-group differences in all baseline characteristics, except for leg press endurance (Table 1).

**Table 1:** Baseline characteristics of the participants.

Variable	3 h (*n* = 16)	immediate (*n* = 15)	*p*-value
**Anthropometry, body composition, and training experience**			
Age (year)	24.8 ± 5.8	24.6 ± 3.6	0.877
Body mass (kg)	77.8 ± 11.2	78.4 ± 11.9	0.893
BMI (kg m^−2^)	24.5 ± 3.6	24.3 ± 3.2	0.858
FM (%)	15.8 ± 9.1	13.3 ± 7.9	0.435
SMM (kg)	35.43 ± 3.72	36.68 ± 4.11	0.385
Training experience (year)	2.6 ± 1.6	3.4 ± 2.9	0.421
**Biochemical markers**			
Creatinine (mg/dL)	1.11 ± 0.15	1.18 ± 0.17	0.217
Urea (mg/dL)	24.6 ± 10.5	26.7 ± 8.2	0.540
AST (U/L)	29.8 ± 14.7	26 ± 11	0.423
ALT (U/L)	26.9 ± 10.2	30.7 ± 12.5	0.950
GGT (U/L)	21.6 ± 8.5	16.4 ± 5.7	0.054
HDL (mg/dL)	43 ± 7	44.4 ± 8.1	0.611
LDL (mg/dL)	82 ± 23	87.4 ± 23.8	0.527
Cholesterol (mg/dL)	149.1 ± 27.2	155.8 ± 27.1	0.504
**Muscular performance**			
Leg press strength (kg)	378.8 ± 119.8	376.7 ± 111.9	0.959
Leg press endurance (r)	15 ± 4.3	11.2 ± 3.3	0.012
Chest press strength (kg)	114 ± 23	119.4 ± 31	0.585
Chest press endurance (r)	10.5 ± 2.1	9.9 ± 1.2	0.386
Vertical jump (cm)	48 ± 8.4	49.2 ± 5.6	0.613
Australian pull-up (r)	19.5 ± 6.8	23.8 ± 8.5	0.137

Values are presented as mean ± standard deviation. BMI, body mass index; FM, fat mass; GGT, gamma-glutamyl transferase; AST, aspartate aminotransferase; ALT, alanine aminotransferase; HDL, high-density lipoprotein; LDL, low-density lipoprotein; y, year; cm, centimeter; kg, kilogram; kg m^−2^, kilogram-meter-2; g, gram; %, percentage; r, repetition; u/L, unit/liter; mg/dL, milligrams*/*deciliter.

### Body composition

**Figure 1:**
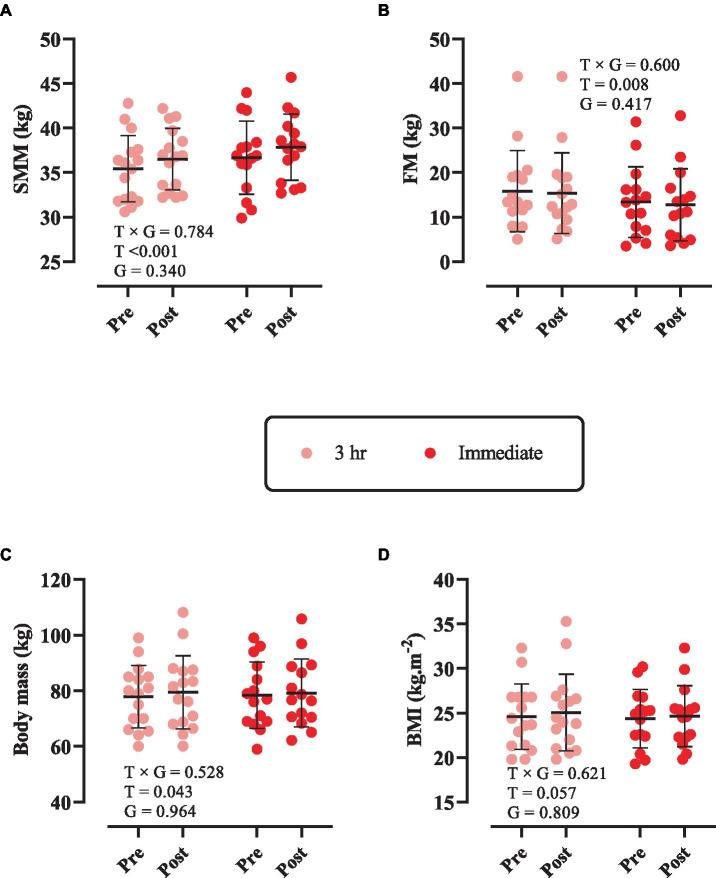
Changes in body composition throughout the intervention. **(A)** Skeletal muscle mass (SMM); **(B)** fat mass (FM); **(C)** body mass; and **(D)** body mass index (BMI). T × G, time × group; T, time; G, group. Error bars represent standard deviation.

Changes in body composition throughout the intervention are shown in Figure 1. There was only a significant main effect of time for SMM (*p* < 0.0001). SMM [3 h = 1.07 kg (95% CI = 0.45 to 1.69, *p* < 0.0001) and immediate = 1.18 kg (95% CI = 0.53 to 1.82, *p* < 0.0001), Figure 1A] significantly increased from pre to post. However, FM (Figure 1B), body mass (Figure 1C), and BMI (Figure 1D) remained unchanged (*p* > 0.05).

### Biochemical markers

**Figure 2:**
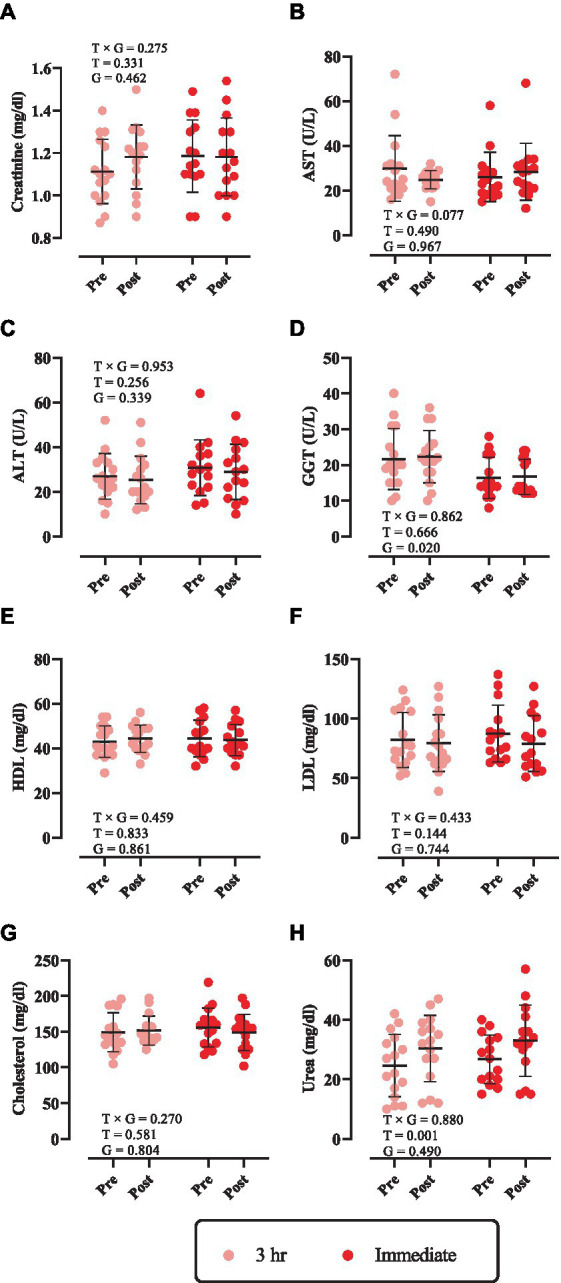
Changes in biochemical markers throughout the intervention. **(A)** Creatinine; **(B)** aspartate transaminase (AST); **(C)** alanine transaminase (ALT); **(D)** gamma-glutamyl transferase (GGT); **(E)** high-density lipoprotein (HDL); **(F)** low-density lipoprotein (LDL); **(G)** cholesterol; and **(H)** urea. T × G, time × group; T, time; G, group. Error bars represent standard deviation.

Changes in biochemical markers throughout the intervention are shown in Figure 2. There was no significant time × group interaction nor main effect of time for creatinine, AST, ALT, GGT, HDL, LDL, and cholesterol (Figures 2A–F; *p* > 0.05). However, a significant main effect of time was observed for urea [3 h = 5.75 mg/dL (95% CI = 0.17 to 11.3, *p* = 0.0423) and immediate = 6.26 mg/dL (95% CI = 0.51 to 12.02, *p* = 0.0311), Figure 2G].

### Muscular performance

**Figure 3:**
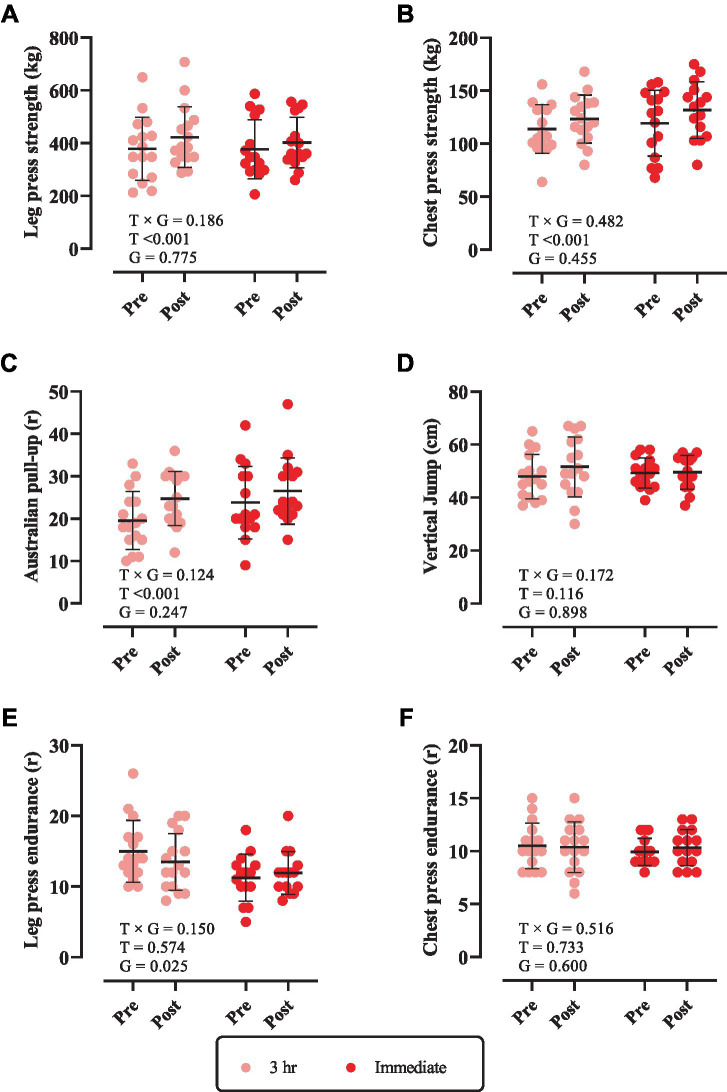
Changes in muscular performance throughout the intervention. **(A)** Leg press strength; **(B)** chest press strength; **(C)** Australian pull-up; **(D)** vertical jump; **(E)** leg press endurance; and **(F)** chest press endurance. T × G, time × group; T, time; G, group. Error bars represent standard deviation.

Changes in muscular performance throughout the intervention are shown in Figure 3. There was a significant main effect of time for leg press strength, chest press strength, and Australian pull-up (*p* < 0.0001). Leg press strength [3 h = 44 kg (95% CI = 21.7 to 66.3, *p* = 0.0001) and immediate = 25.60 kg (95% CI = 2.56 to 48.63, *p* = 0.0274), Figure 3A], chest press strength [3 h = 9.37 kg (95% CI = 2.55 to 16.19, *p* = 0.0059) and immediate = 12.33 kg (95% CI = 5.293 to 19.37, *p* = 0.0006), Figure 3B] and Australian pull-up [3 h = 5.18 *r* (95% CI = 2.64 to 7.73, *p* < 0.0001) and immediate = 2.73 *r* (95% CI = 0.10 to 5.36, *p* = 0.0407), Figure 3C] significantly increased from pre to post. However, there was no time × group interaction nor main effect of time for vertical jump, leg press endurance, and chest press endurance (*p* > 0.05).

### Training volume

**Figure 4:**
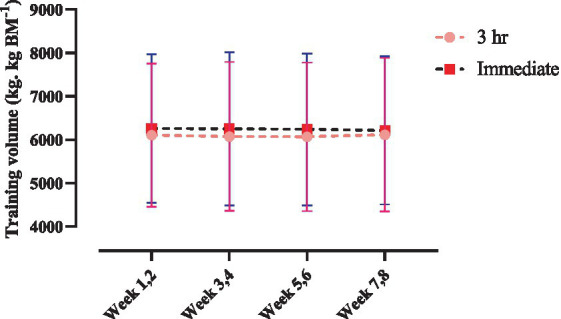
Changes in relative training volume throughout the intervention.

Changes in relative training volume throughout the intervention are shown in Figure 4. There was no time × group interaction nor main effect of time (*p* > 0.05).

### Dietary assessments

Changes in dietary intakes throughout the intervention are shown in Table 2. There was only a significant difference at baseline for relative carbohydrate intake (*p* = 0.049). A significant main effect of time was observed for protein intake [3 h = 0.96 g kg^−1^ d^−1^ kg (95% CI = 0.74 to 1.18, *p* < 0.0001) and immediate = 0.79 g kg^−1^ d^−1^ (95% CI = 0.56 to 1.01, *p* < 0.0001)]. However, there was no time × group interaction nor main effect of time for energy, fat, or carbohydrates (*p* > 0.05).

**Table 2:** Average dietary intake at baseline and throughout the 8 weeks training intervention.

	Time		*p*-value		
	Pre	Post	T × G	T	G
**Relative energy (kcal kg**^**−1**^ **d**^**−1**^**)**					
3 h	27.20 ± 5.52	26 ± 3.88	0.693	0.015	0.055
Immediate	30.18 ± 3.72	28.54 ± 2.88			
**Absolute energy (kcal d**^**−1**^**)**					
3 h	2115.26 ± 512.95	2072.42 ± 508.05	0.669	0.211	0.180
Immediate	2346.46 ± 335.05	2259.89 ± 395.54			
**Relative protein (g kg**^**−1**^ **d**^**−1**^**)**					
3 h	1.07 ± 0.38	2.03 ± 0.06^*^	0.215	<0.001	0.408
Immediate	1.22 ± 0.38	2.01 ± 0.12^*^			
**Absolute protein (g d**^**−1**^**)**					
3 h	83.62 ± 33.53	161.52 ± 26.33^*^	0.267	<0.001	0.595
Immediate	95.16 ± 32.69	159.26 ± 23.73^*^			
**Relative carbohydrate (g kg**^**−1**^ **d**^**−1**^**)**					
3 h	3.91 ± 1.09	3.70 ± 0.94	0.715	0.012	0.033
Immediate	4.69 ± 1	4.41 ± 0.76			
**Absolute carbohydrate (g d**^**−1**^**)**					
3 h	305.06 ± 98.35	296.77 ± 102.95	0.647	0.102	0.088
Immediate	364.74 ± 79.43	350.17 ± 79.67			
**Relative fat (g kg**^**−1**^ **d**^**−1**^**)**					
3 h	0.80 ± 0.23	0.76 ± 0.19	0.507	0.054	0.361
Immediate	0.72 ± 0.22	0.70 ± 0.18			
**Absolute fat (g d**^**−1**^**)**					
3 h	62.27 ± 19.16	59.81 ± 16.31	0.598	0.312	0.396
Immediate	56.31 ± 16.86	55.53 ± 15.81			

^*^p < 0.05 different from baseline.

### Correlations

**Figure 5:**
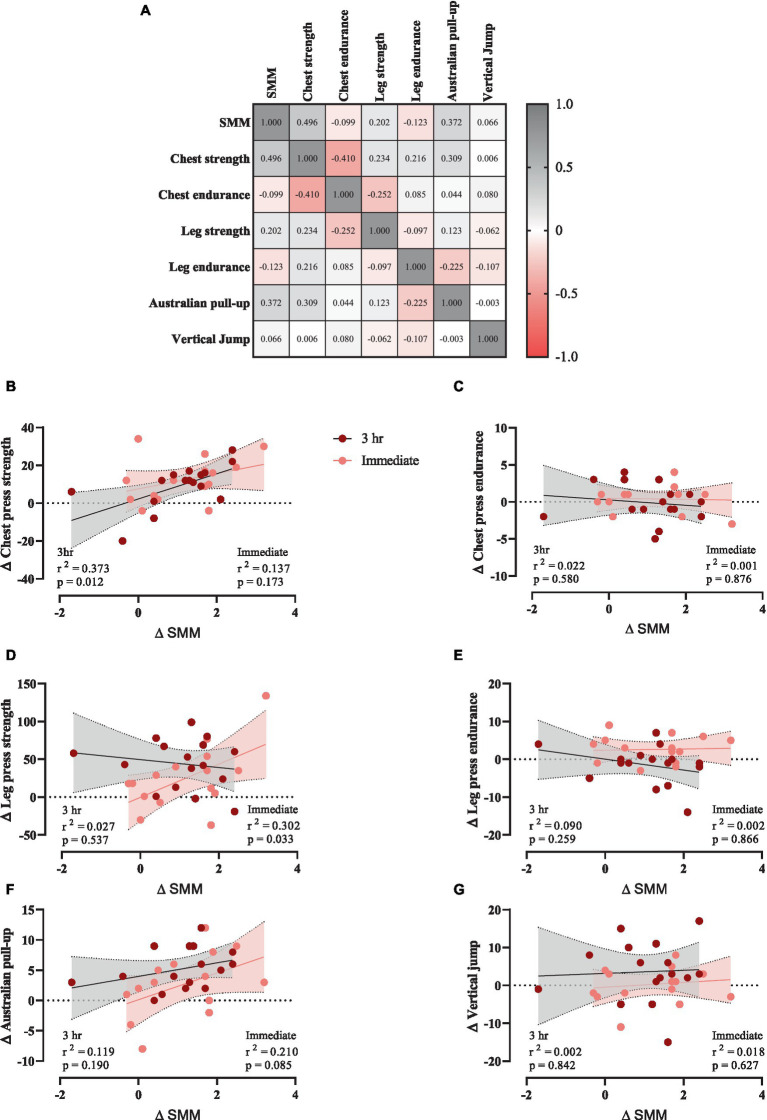
**(A)** Correlation matrix of Δ SMM and performance variables, *r* values are shown. The key indicates the magnitude of *r* (red = −1 or 1, grey = 0). **(B–G)** linear regression (Pearson’s) of Δ (performance) as a function of Δ SMM (kg).

To investigate any potential relationships between training-induced changes in SMM (Δ SMM) and changes in muscular performance (Δ performance variable, independently of 3 h or immediate group), a correlation matrix was generated (Figure 5A). Chest press strength (Figure 5B) and Australian pull-up (Figure 5F) showed moderate positive relationships with Δ SMM, while chest (Figure 5C) and leg press endurance (Figure 5E) showed weak negative relationships. However, leg press strength (Figure 5D) and vertical jump (Figure 5G) showed weak positive relationships. Data were examined using the extra sum-of-squares *F* test to determine which of two equations (models) fits best, with linear regression of individual Δ (performance variable) as a function of Δ SMM. Results showed that chest press strength in 3 h group showed a significant correlation with Δ SMM. *p* and *r*^2^ values are shown.

## Discussion

In this study, we assessed the influence of protein timing on muscle performance and body composition. Our findings indicate that, irrespective of timing, protein supplementation significantly enhanced muscle performance in resistance-trained males. Comparative analysis between the two protein timing groups revealed no significant differences in muscular performance or body composition parameters; both groups equally improved SMM and muscular performance. This improvement highlights that the training paradigm was successful in enhancing performance and body composition variables for both groups, underlining that both groups did successfully undergo a training program. These findings suggest that protein timing may not have effects on indices such as muscular performance and body composition. To the best of our knowledge, this study presents the most up-to-date evidence on the impact of protein timing supplementation on muscle performance and body composition.

Protein supplementation is important for muscle growth and body composition in athletes. According to a study by Cintineo et al. (30), there is robust evidence that consuming protein pre-and/or post-workout induces a significant rise in MPS. Total daily caloric and protein intake over the long term play the most crucial dietary roles in facilitating adaptations to exercise; however, once these factors are accounted for, it appears that peri-exercise protein intake, particularly in the post-training period, plays a potentially useful role in terms of optimizing physical performance and positively influencing the subsequent recovery processes for both resistance training and endurance exercise (31). Another systematic review suggests that as the duration, frequency, and volume of resistance training increase, protein supplementation may promote muscle hypertrophy and enhance gains in muscle strength in both untrained and trained individuals (32). Evidence also suggests that protein supplementation may accelerate gains in both aerobic and anaerobic power (32). The impact of protein timing on muscle performance and body composition remains a topic of debate, with ongoing research exploring how the timing of protein supplementation can influence its effects.

According to the International Society of Sports Nutrition, nutrient timing strategies that involve changing the distribution of intermediate-sized protein doses (20–40 g or 0.25–0.40 g/kg/dose) every three to 4 h best supports increased MPS rates across the day and favorably enhances body composition and physical performance outcomes (33). We observed a positive impact of peri-exercise protein intake as well as 3 h before and after exercise protein intake on muscle performance and body composition. There is evidence that peri-exercise protein intake can have a positive effect on skeletal muscle mass, fat-free mass, fat-free percentage, muscle strength, and muscle endurance. According to the International Society of Sports Nutrition, an acute exercise stimulus, particularly resistance exercise, and protein intake both stimulate MPS and are synergistic when protein intake occurs before or after resistance exercise (8). The combination of protein intake and resistance exercise is the most efficient strategy to promote skeletal muscle hypertrophy and remodeling. However, other protein intake variables should also be considered. The amount, type, and source of proteins, as well as the timing of intake and spreading over the whole day (34). In the context of morning-evening protein intake, Kim et al. (35) found that supplementation of protein at breakfast rather than at dinner and lunch is effective on skeletal muscle mass in older adults. The study showed that the higher the ratio of morning protein intake relative to the total protein intake, the better the muscle mass and handgrip strength. In another, Snijders et al. found that pre-sleep protein intake can have a positive impact on the skeletal muscle’s adaptive response to exercise. Protein ingested prior to sleep is effectively digested and absorbed during overnight sleep, thereby increasing overnight MPS rates. Given that the current study did not identify any statistically significant differences between groups regarding muscular performance and SMM, it is critical to emphasize that any potential impact of protein timing on muscle hypertrophy, if it exists, seems to be relatively minor (36–38). This phenomenon could potentially be attributed to a wider temporal scope of the anabolic window than traditionally hypothesized (extending over several hours), a concept that has been previously suggested (38). Consequently, the overall daily protein intake is unquestionably the most crucial determinant in facilitating muscle growth induced by exercise (37), as seen in the current study with no differences in protein doses between groups.

In the context of protein timing, we should consider some major issues that can be helpful in muscle performance and body composition alteration. Different muscles have varying proportions of muscle fiber types (slow-twitch vs. fast-twitch). Endurance activities tend to involve slow-twitch fibers, while grip strength exercises might engage fast-twitch fibers (39). The timing of protein intake could affect these fiber types differently. In addition, upper-body endurance activities require a sustained energy supply, whereas grip strength exercises demand rapid bursts of energy (40). Protein timing could influence how these energy demands are met during the activities. Furthermore, the differences in muscle groups and energy demands might lead to varied rates of muscle recovery and adaptation (41). The timing of protein intake might influence how well the muscles recover and adapt to the specific demands of the exercises. Moreover, each participant’s body may respond differently to protein timing based on their genetics, training history, and overall nutritional status.

Our study had some limitations. First, the participants in our study had varying levels of fitness, training experience, and genetics. This variability could influence the response to protein supplementation and complicate the interpretation of the results. Second, the duration of our study was 8 weeks, which might impact the outcomes observed. Third, various factors outside of the study design, such as participants’ sleep patterns, stress levels, and other lifestyle factors, could impact muscle performance and body composition. Fourth, the choice of outcome measures, such as body composition assessment methods, can influence the results. Utilizing dual X-ray absorptiometry (DEXA) to determine body composition would be preferable for future research. Fifth, despite implementing progressive overload in our training intervention, our statistical analysis revealed no significant time effect on training volume. However, progressive overload is evident through the significant increases in strength observed in both groups. Finally, the results of our study might be limited in their applicability to different populations, such as elite athletes.

## Conclusion

Protein supplementation enhances muscular performance and SMM in resistance-trained males, irrespective of intake time. These findings provide further evidence to the theory that the traditionally postulated “anabolic window” may not be as narrow as commonly proposed (38), at least in trained participants. Consequently, the total daily protein intake appears to be the primary factor in facilitating muscle growth induced by exercise. Future research could delve into the impact of protein timing on different populations and employ more standardized outcome measures.

## Data availability statement

The raw data supporting the conclusions of this article will be made available by the authors, without undue reservation.

## Ethics statement

The studies involving humans and all protocols were approved by the Research Ethics Committees of the Sport Sciences Research Institute, Tehran, Iran (IR.SSRC.REC.1403.059). The studies were conducted in accordance with the local legislation and institutional requirements. The participants provided their written informed consent to participate in this study.

## Author contributions

ML: Conceptualization, Methodology, Writing – original draft, Writing – review & editing, Supervision, Funding acquisition. RB: Software, Validation, Investigation, Writing – review & editing, Supervision and Project administration. HG: Data curation, Resources, Writing – review & editing. BC: Conceptualization, Methodology, Writing – original draft, Writing – review & editing. AW: Methodology, Writing – review & editing. AS: Conceptualization, Formal analysis, Supervision, Writing – review & editing. MS: Data curation, Project administration, Writing – review & editing. FD: Conceptualization, Funding acquisition, Resources, Writing – review & editing. All authors have read and agreed to the published version of the manuscript.
