# Correction: Timing matters? The effects of two different timing of high protein diets on body composition, muscular performance, and biochemical markers in resistance-trained males

**DOI:** 10.3389/fnut.2025.1675832

**Published:** 2025-08-14

**Authors:** Mohammadyasin Lak, Reza Bagheri, Hamid Ghobadi, Bill Campbell, Alexei Wong, Amin Shahrbaf, Mohammad Shariatzadeh, Fred Dutheil

**Affiliations:** ^1^Sport Sciences Research Institute of Iran, Tehran, Iran; ^2^Department of Exercise Physiology, University of Isfahan, Isfahan, Iran; ^3^Department of Exercise Physiology, Ferdowsi University of Mashhad, Mashhad, Iran; ^4^Performance and Physique Enhancement Laboratory, University of South Florida, Tampa, FL, United States; ^5^Department of Health and Human Performance, Marymount University, Arlington, TX, United States; ^6^Faculty of Medicine, Shahid Beheshti University of Medical Sciences, Tehran, Iran; ^7^Université Clermont Auvergne CNRS, LaPSCo, Physiological and Psychosocial Stress, CHU Clermont-Ferrand, University Hospital of Clermont-Ferrand, Preventive and Occupational Medicine, Clermont-Ferrand, France

**Keywords:** exercise, dietary protein, nutrition, muscle hypertrophy, strength

In the published article, there was an error in **Methods**, “*Participants*”. The first sentence was incorrectly written as, “All protocols were approved by the institutional review board of Shahid Beheshti University of Medical Sciences, Tehran, Iran, carried out in accordance with the Declaration of Helsinki and registered at clinicaltrials.gov (NCT05544955).” The correct sentence should have been written as, “All protocols were approved by the Research Ethics Committees of the Sport Sciences Research Institute, Tehran, Iran, carried out in accordance with the Declaration of Helsinki and registered at clinicaltrials.gov (NCT05544955).”

In the published article, there was an error in the **Ethics Statement**. The statement was incorrectly written as, “The studies involving humans and all protocols were approved by the institutional review board of Shahid Beheshti University of Medical Sciences, Tehran, Iran (IR.SBMU.MSP.REC.1400.708). The correct statement should have been written as, “The studies involving humans and all protocols were approved by the Research Ethics Committees of the Sport Sciences Research Institute, Tehran, Iran (IR.SSRC.REC.1403.059).”

The original article has been updated.

